# Supplementary material for: Genome-Wide Analysis of the UDP-Glycosyltransferase Family Reveals Its Roles in Coumarin Biosynthesis and Abiotic Stress in Melilotus albus
Source: Int J Mol Sci. 2021 Oct 6;22(19):10826. doi: 10.3390/ijms221910826 (PMC8509628; doi:10.3390/ijms221910826)
Supplement: Supplementary file 1 [file ijms-22-10826-s001.zip › ijms-1391494-supplementary.pdf]

Genome-wide analysis of the UDP-glycosyltransferase family reveals its roles in coumarin biosynthesis and abiotic stress in *Melilotus albus*

Supplementary Materials

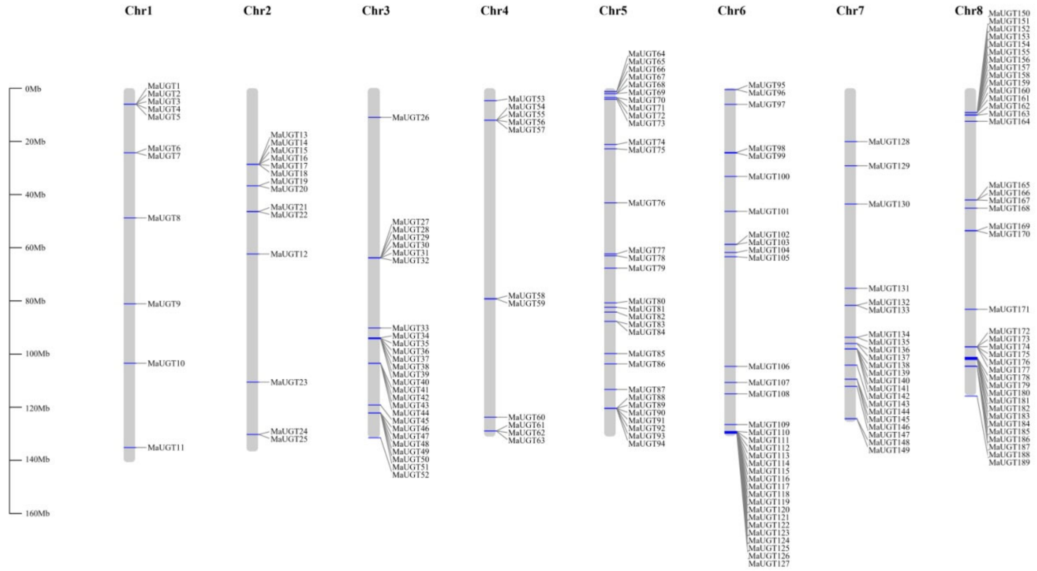

Supplementary Figure S1. Chromosomal distribution of 189 *UGT* genes identified in the *M. albus* genome. Eight chromosomes are indicated in gray columns. All predicated *MaUGT* genes are indicated by a blue stick on the column and the locus numbers of *MaUGT* genes are indicated on the right side.

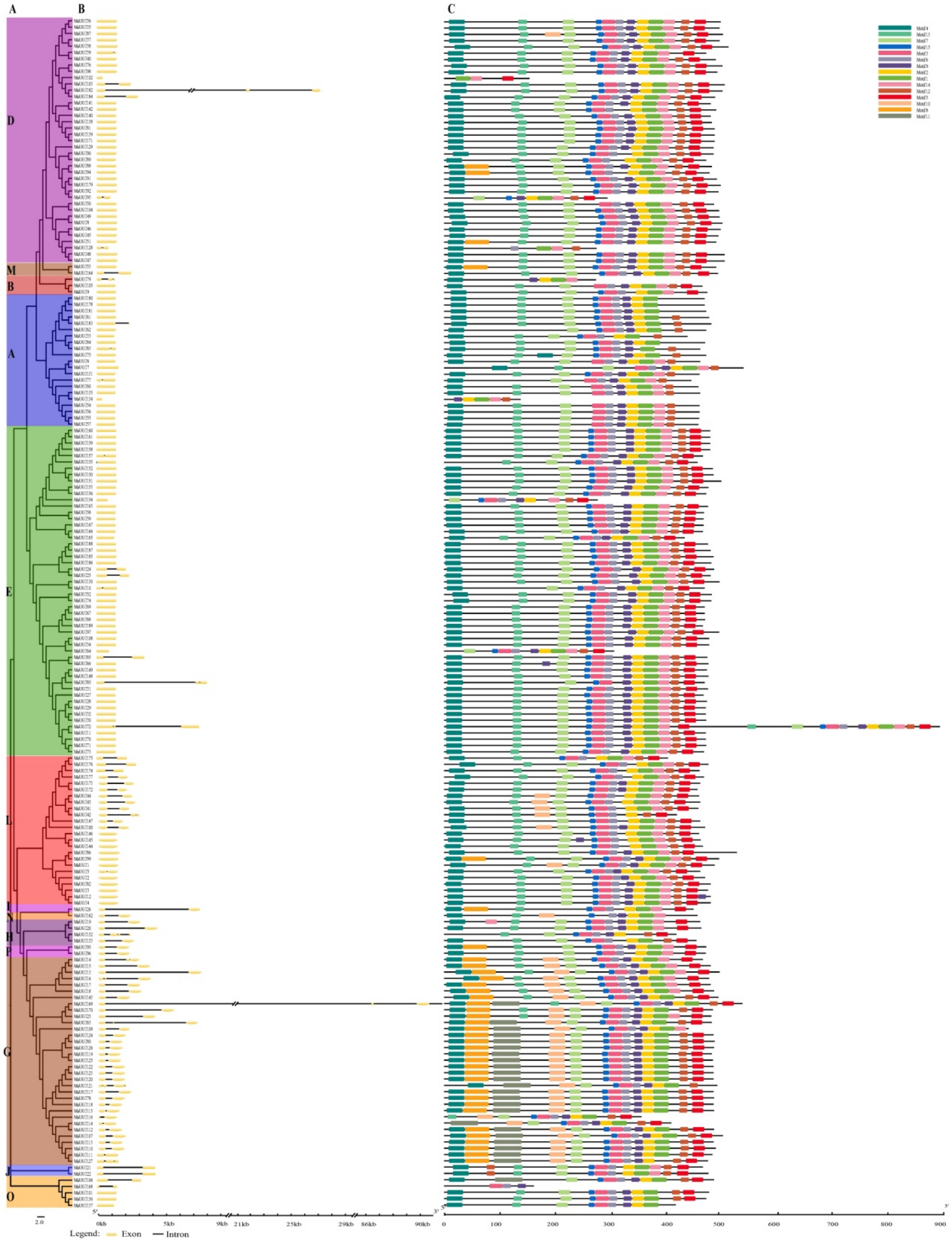

Supplementary Figure S2. Phylogenetic and gene structure analysis of *UGT* genes in *M. albus*: (A), The phylogenetic tree was constructed with MEGA 7.0 using the neighbor-joining (NJ) method with 1000 bootstrap replicates based on a multiple alignment of amino acid sequences of *UGT* genes. The subgroup was classified and marked by different color; (B), Gene structures of *MaUGTs*. CDS exon indicated by yellow boxes, intron indicated by black line; (C), Protein motif. Schematic diagram of the conserved motifs in the UGT proteins, which were elucidated using MEME. Each motif is represented by a number in the colored box. The black lines represent the non-conserved sequences.

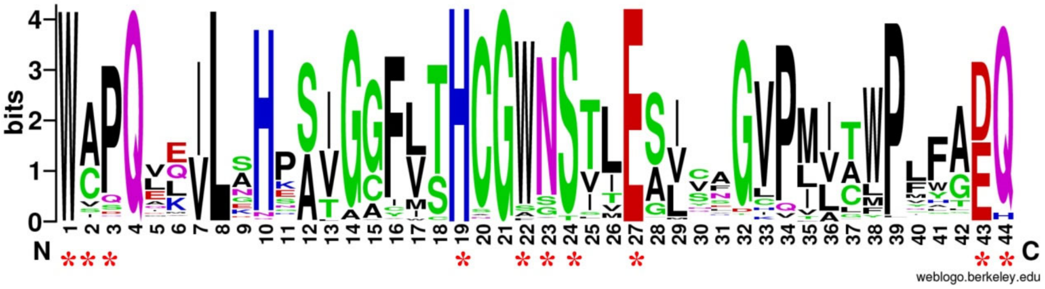

Supplementary Figure S3. Web logo representing the PSPG-box motif of MaUGTs. The asterisks indicate the key amino acid residues interacting directly with the sugar donor. The logo consists of stacks of symbols, one stack for each position in the sequence. The overall height of the stack indicates the sequence conservation at that position, while the height of symbols within the stack indicates the relative frequency of each amino acid at that position.

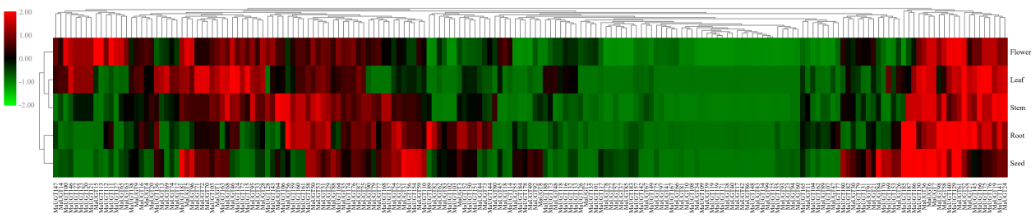

Supplementary Figure S4. Hierarchical clustering analysis of transcript levels of *UGT* genes in different tissues of *M. albus*. Data were retrieved from transcriptome datasets, and the clustering was performed using TBtools. The heat map shows the relative transcript level of *MaUGT* genes in various tissues. The color scale (–2 to 2 in green to red color) represents Z-score-normalized gene expression. Dendrograms along the top and left sides of the heat map indicate the hierarchical clustering of genes and tissues.

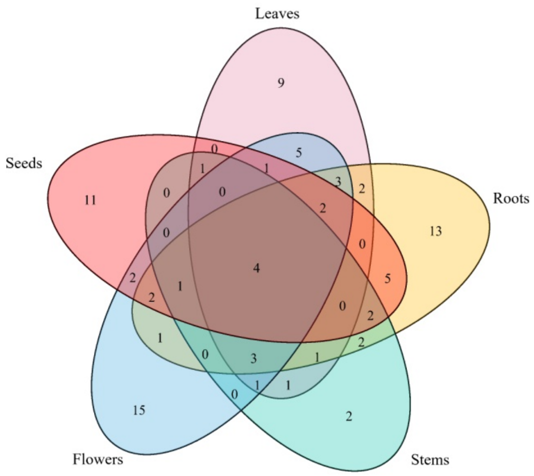

Supplementary Figure S5. Venn diagram showing overlap of highly expressed *MaUGT* genes (FPKM ≥10) in different tissues.

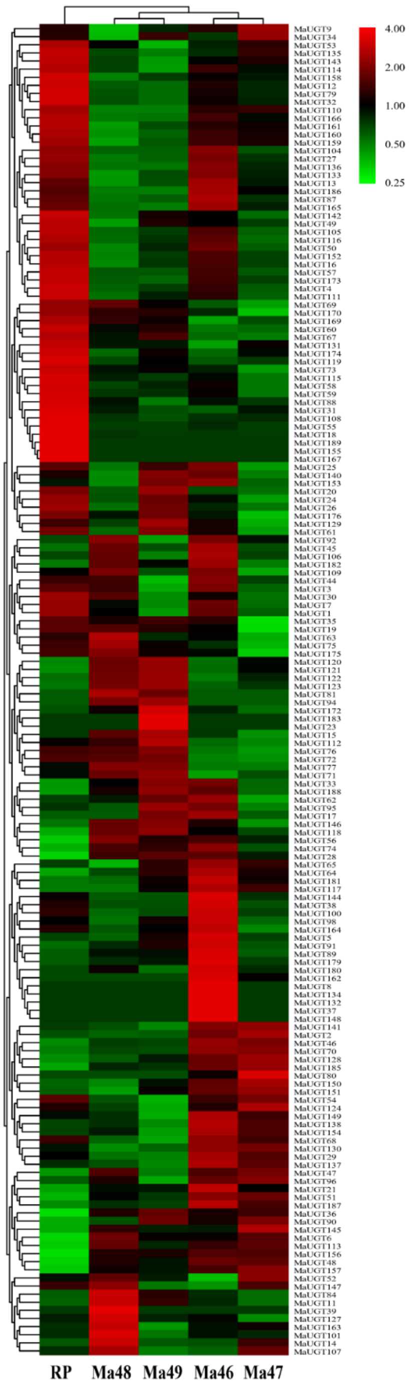

Supplementary Figure S6. Expression of 189 *MaUGT* genes among five *M. albus* genotypes. Data were retrieved Figure 46. Ma47, Ma48 and Ma49) and the recurrent male parent (RP). The color scale (–2 to 2 in green to red color) represents Z-score-normalized gene expression. Dendrograms along the top and left sides of the heat map indicate the hierarchical clustering of NILs and genes.

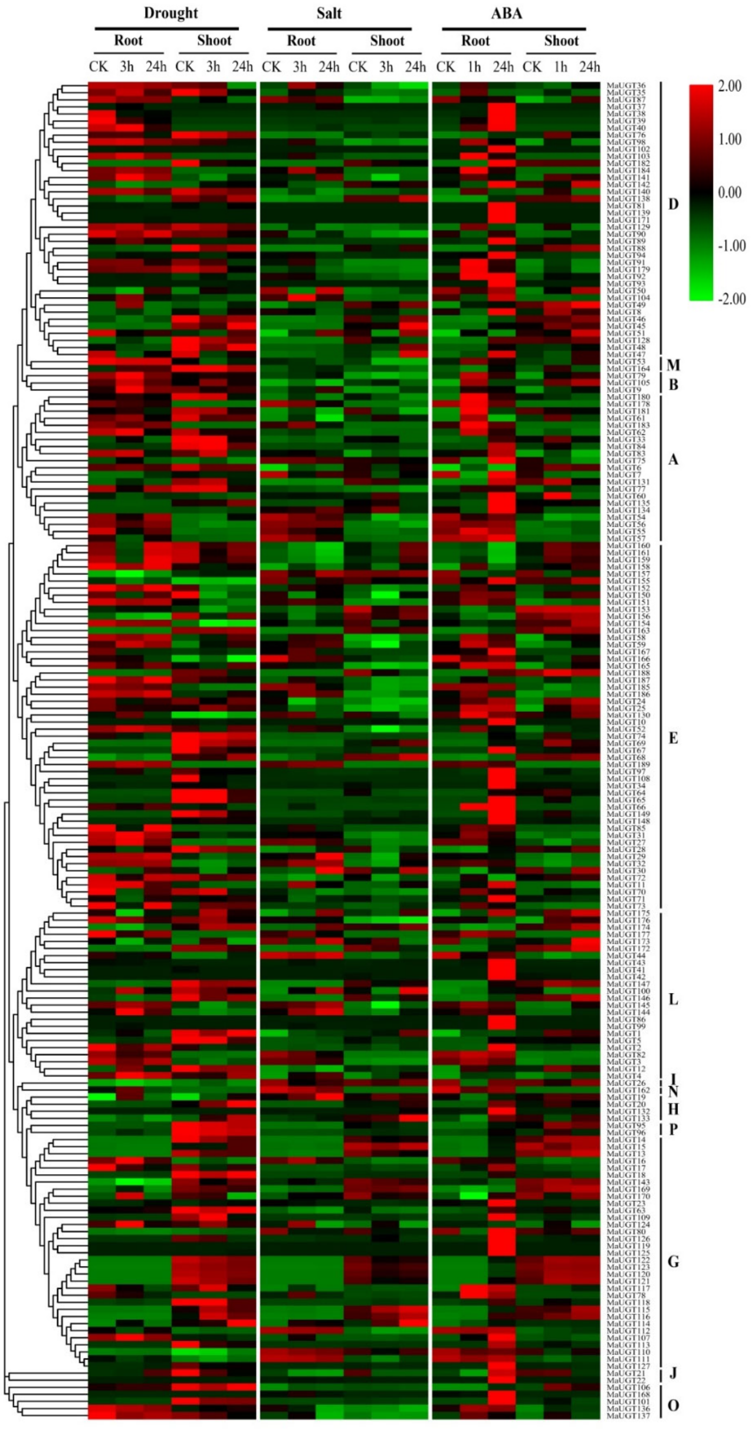

Supplementary Figure S7. Expression of 189 *MaUGT* genes in response to drought, salt and ABA treatments. Data were retrieved from transcriptome datasets, and the clustering was performed using TBtools. The heat map shows the relative transcript level of *MaUGT* genes under drought, salt and ABA stresses. The color scale (–2 to 2 in green to red color) represents Z-score-normalized gene expression. Dendrograms along the left side of the heat map indicate the phylogenetic relationship of 189 *UGT*s from *M. albus*. The expression values (FPKM) were normalized.

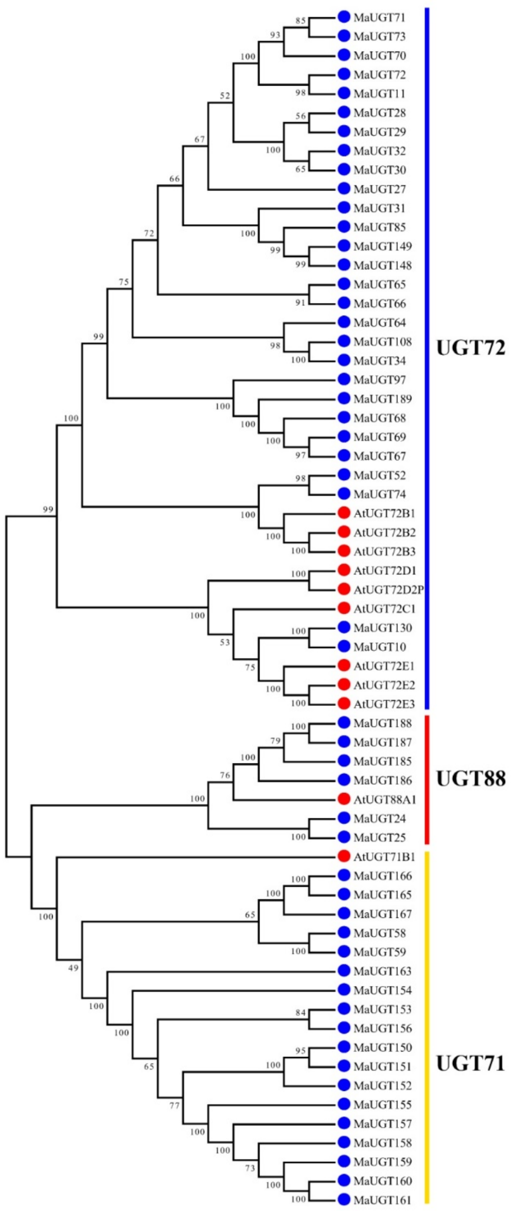

Supplementary Figure S8. Phylogenetic analysis of the UGTs from *M. albus* in Group E. A total of 52 UGT proteins from *M. albus*, 11 UGTs from Arabidopsis were used to construct the neighbour-joining tree using the program MEGA 7.0. All protein sequences were full length, and the bootstrap values of 1000 replicates were calculated at each node.

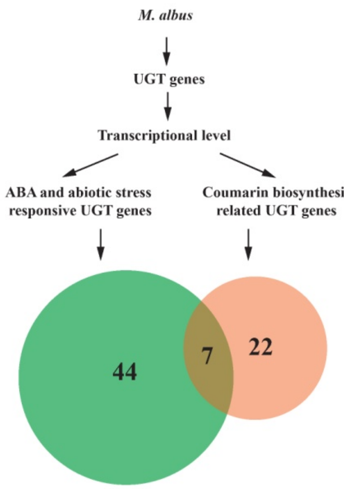

Supplementary Figure S9. Model of functional divergence of coumarin biosynthesis-related and stress-responsive *MaUGT* genes in *M. albus*.

Table S1. Basic information regarding the UGT proteins in *M. Albus*.

Gene name	Locus ID	Protein length (aa)	Mw (k Da)	p Is	GRAVY	Localization
MaUGT1	Malbus0100511.1	486	54.41349	5.01	-0.126	Cytoplasmic/PlasmaMembrane
MaUGT2	Malbus0100512.1	469	51.90963	6.24	-0.066	Cytoplasmic
MaUGT3	Malbus0100513.1	474	52.77105	5.75	0.014	Cytoplasmic
MaUGT4	Malbus0100514.1	479	53.69594	5.57	-0.076	Cytoplasmic
MaUGT5	Malbus0100516.1	470	51.87718	5.75	-0.101	Cytoplasmic
MaUGT6	Malbus0101841.1	460	52.25249	5.94	-0.061	Cytoplasmic
MaUGT7	Malbus0101842.1	538	61.24173	7.95	-0.152	Cytoplasmic/Mitochondrial
MaUGT8	Malbus0103152.1	494	55.75633	5.53	-0.202	Cytoplasmic
MaUGT9	Malbus0103939.1	473	52.82342	5.93	-0.081	PlasmaMembrane
MaUGT10	Malbus0104457.1	498	55.15321	5.35	-0.051	PlasmaMembrane/Chloroplast/Cytoplasmic
MaUGT11	Malbus0105782.1	470	51.81544	5.94	-0.078	Cytoplasmic
MaUGT12	Malbus0203779.1	479	53.75091	6.29	-0.103	Cytoplasmic
MaUGT13	Malbus0202288.1	495	56.37961	5.71	-0.269	Cytoplasmic
MaUGT14	Malbus0202289.1	465	52.82146	5.46	-0.239	Cytoplasmic
MaUGT15	Malbus0202290.1	545	62.39654	6.18	-0.29	Cytoplasmic
MaUGT16	Malbus0202291.1	510	57.98071	6.37	-0.272	Cytoplasmic
MaUGT17	Malbus0202293.1	479	54.59965	6.25	-0.288	Cytoplasmic
MaUGT18	Malbus0202295.1	485	54.76284	5.99	-0.226	Cytoplasmic
MaUGT19	Malbus0202780.1	460	51.59051	6.04	-0.073	Cytoplasmic
MaUGT20	Malbus0202781.1	956	109.58556	8.83	-0.229	PlasmaMembrane
MaUGT21	Malbus0203198.1	465	53.10869	8.38	-0.05	PlasmaMembrane
MaUGT22	Malbus0203199.1	475	54.33515	7.19	-0.011	PlasmaMembrane
MaUGT23	Malbus0204872.1	480	53.76961	5.43	-0.235	Cytoplasmic
MaUGT24	Malbus0205401.1	485	53.8086	5.95	-0.029	PlasmaMembrane/Chloroplast
MaUGT25	Malbus0205402.1	479	53.68671	6.64	-0.088	Chloroplast/Mitochondrial
MaUGT26	Malbus0300313.1	448	50.88323	5.33	-0.225	Cytoplasmic/PlasmaMembrane
MaUGT27	Malbus0301445.1	466	51.92114	5.61	0.013	Cytoplasmic/Chloroplast
MaUGT28	Malbus0301447.1	472	52.30156	6.24	0.001	Cytoplasmic/Chloroplast

MaUGT29	Malbus0301448.1	470	51.74687	6.11	0.006	Cytoplasmic/Chloroplast
MaUGT30	Malbus0301450.1	471	52.14721	5.45	0.001	Cytoplasmic
MaUGT31	Malbus0301451.1	474	52.50418	5.25	0.031	PlasmaMembrane
MaUGT32	Malbus0301452.1	470	51.88694	5.82	-0.029	Cytoplasmic
MaUGT33	Malbus0302202.1	437	49.6352	5.48	-0.165	Cytoplasmic
MaUGT34	Malbus0302367.1	460	50.93661	5.44	-0.003	Cytoplasmic/Chloroplast/PlasmaMembrane
MaUGT35	Malbus0302374.1	495	56.09471	5.78	-0.395	Cytoplasmic
MaUGT36	Malbus0302375.1	497	56.31319	6.24	-0.444	Cytoplasmic
MaUGT37	Malbus0302376.1	495	55.96096	5.63	-0.284	Cytoplasmic
MaUGT38	Malbus0302377.1	511	57.70486	5.41	-0.252	Cytoplasmic
MaUGT39	Malbus0302378.1	482	54.36728	6.45	-0.261	Cytoplasmic
MaUGT40	Malbus0302381.1	488	55.33427	5.96	-0.257	PlasmaMembrane/Cytoplasmic
MaUGT41	Malbus0302900.1	457	51.80986	5.84	-0.13	Cytoplasmic
MaUGT42	Malbus0302902.1	418	47.37175	5.99	-0.162	Cytoplasmic
MaUGT43	Malbus0302903.1	460	52.11709	6.09	-0.12	Cytoplasmic
MaUGT44	Malbus0302904.1	458	52.19325	5.77	-0.149	Cytoplasmic
MaUGT45	Malbus0304042.1	495	55.58965	6.1	-0.082	Cytoplasmic
MaUGT46	Malbus0304268.1	497	55.77377	6.3	-0.083	Cytoplasmic
MaUGT47	Malbus0304269.1	504	56.82578	6.06	-0.167	Cytoplasmic
MaUGT48	Malbus0304270.1	504	57.02021	6.12	-0.144	Cytoplasmic
MaUGT49	Malbus0304271.1	482	55.07496	8.41	-0.292	Cytoplasmic
MaUGT50	Malbus0304273.1	399	44.83307	6.24	-0.185	Cytoplasmic
MaUGT51	Malbus0304274.1	489	55.434	5.67	-0.148	Cytoplasmic
MaUGT52	Malbus0305129.1	481	53.01007	5.73	-0.091	Cytoplasmic
MaUGT53	Malbus0400484.1	489	54.75689	6.16	-0.092	PlasmaMembrane/Cytoplasmic
MaUGT54	Malbus0400922.1	459	51.37233	5.74	-0.084	Cytoplasmic
MaUGT55	Malbus0400924.1	458	51.0934	6.12	0.019	Cytoplasmic
MaUGT56	Malbus0400926.1	459	51.28334	6.29	-0.09	Cytoplasmic
MaUGT57	Malbus0400927.1	457	51.19627	6.25	-0.071	Cytoplasmic/Extracellular
MaUGT58	Malbus0402780.1	467	52.67483	5.75	-0.011	PlasmaMembrane
MaUGT59	Malbus0402783.1	466	52.48458	5.76	-0.039	PlasmaMembrane
MaUGT60	Malbus0404514.1	457	51.07441	6.38	0.041	PlasmaMembrane/Cytoplasmic
MaUGT61	Malbus0404910.1	476	53.59931	5.47	-0.246	Cytoplasmic
MaUGT62	Malbus0404911.1	471	53.63553	5.87	-0.243	Cytoplasmic
MaUGT63	Malbus0404914.1	481	53.80484	6.18	0.023	Cytoplasmic
MaUGT64	Malbus0500048.1	478	52.99561	7.12	0.055	PlasmaMembrane
MaUGT65	Malbus0500051.1	473	53.27052	6.96	-0.057	PlasmaMembrane
MaUGT66	Malbus0500052.1	475	52.32977	6.17	0.144	PlasmaMembrane
MaUGT67	Malbus0500081.1	468	51.90695	5.47	0.07	Cytoplasmic/PlasmaMembrane
MaUGT68	Malbus0500085.1	469	52.02519	5.99	-0.009	Cytoplasmic/PlasmaMembrane
MaUGT69	Malbus0500087.1	468	51.91182	5.13	0.016	Cytoplasmic/PlasmaMembrane
MaUGT70	Malbus0500190.1	969	106.64381	5.76	0.022	PlasmaMembrane
MaUGT71	Malbus0500191.1	471	51.75851	5.73	0.031	Cytoplasmic
MaUGT72	Malbus0500192.1	892	98.53298	5.74	-0.039	PlasmaMembrane/Cytoplasmic
MaUGT73	Malbus0500234.1	467	51.70378	6.65	-0.025	Nuclear
MaUGT74	Malbus0501174.1	480	53.61949	5.54	-0.168	Cytoplasmic
MaUGT75	Malbus0501297.1	471	52.78313	6.21	0.013	Cytoplasmic/PlasmaMembrane
MaUGT76	Malbus0501975.1	500	56.36045	5.52	-0.274	Cytoplasmic
MaUGT77	Malbus0502341.1	448	49.75921	5.88	-0.049	Cytoplasmic
MaUGT78	Malbus0502372.1	485	54.74515	5.63	-0.084	PlasmaMembrane

MaUGT79	Malbus0502448.1	274	30.29503	8.35	0.018	PlasmaMembrane
MaUGT80	Malbus0502730.1	498	56.16966	6.18	-0.182	Cytoplasmic
MaUGT81	Malbus0502769.1	486	55.01945	5.51	-0.099	Cytoplasmic
MaUGT82	Malbus0502831.1	479	53.57603	5.98	0.018	Cytoplasmic/PlasmaMembrane
MaUGT83	Malbus0502959.1	460	52.4554	5.59	-0.201	Cytoplasmic
MaUGT84	Malbus0502960.1	469	53.60185	6.04	-0.196	Cytoplasmic
MaUGT85	Malbus0503461.1	468	51.92545	5.37	-0.047	PlasmaMembrane
MaUGT86	Malbus0503647.1	526	59.1497	5.13	-0.251	Cytoplasmic
MaUGT87	Malbus0504231.1	501	56.7014	6	-0.357	Cytoplasmic/PlasmaMembrane
MaUGT88	Malbus0504723.1	481	53.96415	6.36	-0.16	Cytoplasmic
MaUGT89	Malbus0504724.1	471	53.34322	5.91	-0.156	Cytoplasmic/PlasmaMembrane
MaUGT90	Malbus0504725.1	472	53.38619	5.75	-0.209	Cytoplasmic
MaUGT91	Malbus0504726.1	490	54.7576	5.52	-0.211	Cytoplasmic
MaUGT92	Malbus0504727.1	493	55.14888	5.65	-0.255	Cytoplasmic
MaUGT93	Malbus0504728.1	485	54.32568	6.59	-0.143	Cytoplasmic/Chloroplast
MaUGT94	Malbus0504731.1	477	53.69261	6.31	-0.206	Cytoplasmic
MaUGT95	Malbus0600024.1	471	54.09855	5.96	-0.13	Cytoplasmic/PlasmaMembrane/Nuclear
MaUGT96	Malbus0600026.1	470	53.18058	6.55	-0.115	Cytoplasmic/Nuclear
MaUGT97	Malbus0600471.1	494	55.20239	5.65	-0.021	PlasmaMembrane
MaUGT98	Malbus0601344.1	491	55.74319	5.7	-0.221	Cytoplasmic
MaUGT99	Malbus0601358.1	494	55.56028	5.11	-0.19	Cytoplasmic/PlasmaMembrane
MaUGT100	Malbus0601730.1	469	52.25101	5.93	-0.149	Cytoplasmic
MaUGT101	Malbus0602035.1	476	52.61563	6.14	-0.009	Chloroplast
MaUGT102	Malbus0602458.1	152	16.83174	7.87	0.104	Chloroplast/InnerMembrane
MaUGT103	Malbus0602461.1	504	56.94032	5.66	-0.197	Cytoplasmic
MaUGT104	Malbus0602531.1	494	56.09504	6.33	-0.09	PlasmaMembrane/Cytoplasmic
MaUGT105	Malbus0602563.1	464	51.46584	5.68	-0.042	Chloroplast/Cytoplasmic
MaUGT106	Malbus0603554.1	486	54.07476	5.94	-0.019	Chloroplast
MaUGT107	Malbus0603793.1	472	52.90186	5.33	-0.129	Cytoplasmic
MaUGT108	Malbus0603968.1	476	52.76354	5.09	-0.034	Cytoplasmic/Chloroplast
MaUGT109	Malbus0604419.1	482	54.51611	6.39	-0.098	Cytoplasmic/PlasmaMembrane
MaUGT110	Malbus0604552.1	488	55.34073	6.18	-0.14	Cytoplasmic/PlasmaMembrane
MaUGT111	Malbus0604553.1	482	54.64584	5.46	-0.144	Cytoplasmic/PlasmaMembrane
MaUGT112	Malbus0604554.1	475	53.73507	5.94	-0.129	Cytoplasmic
MaUGT113	Malbus0604556.1	484	54.76008	5.5	-0.144	Cytoplasmic
MaUGT114	Malbus0604557.1	475	53.89211	5.88	-0.107	PlasmaMembrane/Cytoplasmic
MaUGT115	Malbus0604558.1	485	54.3193	5.42	-0.122	Cytoplasmic/PlasmaMembrane
MaUGT116	Malbus0604560.1	354	39.73873	5.1	-0.087	PlasmaMembrane/Cytoplasmic
MaUGT117	Malbus0604561.1	484	54.9346	5.79	-0.086	PlasmaMembrane
MaUGT118	Malbus0604562.1	484	54.66519	5.68	-0.105	Cytoplasmic/Nuclear
MaUGT119	Malbus0604563.1	468	52.79766	5.4	-0.175	Cytoplasmic
MaUGT120	Malbus0604567.1	484	54.70284	5.63	-0.212	Cytoplasmic/Nuclear
MaUGT121	Malbus0604568.1	491	55.79644	5.99	-0.116	PlasmaMembrane
MaUGT122	Malbus0604569.1	484	54.70284	5.63	-0.212	Cytoplasmic/Nuclear
MaUGT123	Malbus0604570.1	484	54.70284	5.63	-0.212	Cytoplasmic/Nuclear
MaUGT124	Malbus0604571.1	484	54.35796	6.08	-0.061	Cytoplasmic
MaUGT125	Malbus0604572.1	483	54.56387	5.6	-0.094	PlasmaMembrane/Cytoplasmic
MaUGT126	Malbus0604576.1	485	54.41683	5.39	-0.072	Cytoplasmic
MaUGT127	Malbus0604592.1	464	52.37741	5.5	-0.087	Cytoplasmic
MaUGT128	Malbus0700754.1	249	27.80465	7.69	-0.006	Cytoplasmic

MaUGT129	Malbus0701003.1	484	53.9587	5.63	-0.268	Cytoplasmic
MaUGT130	Malbus0701391.1	495	54.44035	5.27	-0.026	Cytoplasmic/PlasmaMembrane
MaUGT131	Malbus0702099.1	459	52.17247	5.88	0.023	Cytoplasmic/PlasmaMembrane
MaUGT132	Malbus0702329.1	417	46.74997	6.28	-0.006	Cytoplasmic
MaUGT133	Malbus0702333.1	453	51.22901	6.02	-0.044	Cytoplasmic/PlasmaMembrane
MaUGT134	Malbus0703023.1	134	14.54178	5.69	-0.031	Cytoplasmic
MaUGT135	Malbus0703024.1	460	51.49908	5.98	-0.166	Cytoplasmic
MaUGT136	Malbus0703189.1	471	51.91993	5.66	-0.006	Chloroplast
MaUGT137	Malbus0703190.1	456	50.5104	6.02	0.01	Chloroplast
MaUGT138	Malbus0703325.1	481	54.00105	5.57	-0.141	Cytoplasmic
MaUGT139	Malbus0703326.1	486	54.99075	7.28	-0.157	Cytoplasmic
MaUGT140	Malbus0703327.1	479	53.56558	5.68	-0.217	Cytoplasmic
MaUGT141	Malbus0703328.1	479	54.10388	5.71	-0.313	Cytoplasmic/Chloroplast
MaUGT142	Malbus0703329.1	489	55.52557	5.26	-0.292	Cytoplasmic
MaUGT143	Malbus0703818.1	493	55.98357	5.43	-0.217	Cytoplasmic
MaUGT144	Malbus0704244.1	465	52.24629	4.91	-0.191	Cytoplasmic
MaUGT145	Malbus0704245.1	468	52.91364	5.64	-0.169	Cytoplasmic
MaUGT146	Malbus0704247.1	458	51.99662	5.56	-0.168	PlasmaMembrane
MaUGT147	Malbus0704438.1	460	51.63282	6.02	-0.01	PlasmaMembrane/Cytoplasmic
MaUGT148	Malbus0705388.1	475	53.04223	5.57	0.008	PlasmaMembrane
MaUGT149	Malbus0705393.1	472	52.68014	5.05	-0.066	PlasmaMembrane
MaUGT150	Malbus0800594.1	484	53.67646	6.44	-0.145	Nuclear
MaUGT151	Malbus0800595.1	498	55.42735	6.55	-0.207	Cytoplasmic
MaUGT152	Malbus0800596.1	483	53.56639	5.57	-0.08	Cytoplasmic
MaUGT153	Malbus0800597.1	475	52.63635	4.86	0.062	PlasmaMembrane
MaUGT154	Malbus0800599.1	471	52.00247	5	-0.002	Cytoplasmic/PlasmaMembrane
MaUGT155	Malbus0800600.1	429	48.00737	5.87	0.028	PlasmaMembrane
MaUGT156	Malbus0800601.1	471	52.00247	5	-0.002	Cytoplasmic/PlasmaMembrane
MaUGT157	Malbus0800602.1	465	51.09999	6.39	0.064	PlasmaMembrane/Chloroplast
MaUGT158	Malbus0800604.1	478	52.49628	5.34	0.026	Chloroplast/PlasmaMembrane
MaUGT159	Malbus0800606.1	478	52.81376	5.73	0.045	PlasmaMembrane
MaUGT160	Malbus0800607.1	478	52.67971	5.73	0.079	PlasmaMembrane
MaUGT161	Malbus0800608.1	478	52.65166	5.73	0.074	PlasmaMembrane
MaUGT162	Malbus0800655.1	455	51.32556	8.05	-0.034	PlasmaMembrane
MaUGT163	Malbus0800663.1	474	52.07158	5.01	0.031	Cytoplasmic/Chloroplast
MaUGT164	Malbus0800838.1	492	55.01323	5.61	-0.145	Cytoplasmic
MaUGT165	Malbus0802012.1	432	48.51205	5.15	-0.024	Cytoplasmic
MaUGT166	Malbus0802013.1	465	52.21427	5.2	-0.007	Cytoplasmic
MaUGT167	Malbus0802018.1	466	52.00922	6.13	-0.026	Cytoplasmic
MaUGT168	Malbus0802150.1	160	19.0852	8.81	-0.428	Cytoplasmic
MaUGT169	Malbus0802422.1	479	53.83504	5.45	-0.191	Cytoplasmic
MaUGT170	Malbus0802423.1	472	52.98695	5.24	-0.067	Cytoplasmic
MaUGT171	Malbus0803050.1	486	54.99075	7.28	-0.157	Cytoplasmic
MaUGT172	Malbus0803445.1	455	51.38997	5.28	-0.167	Cytoplasmic
MaUGT173	Malbus0803447.1	460	52.14121	5.41	-0.09	Cytoplasmic/PlasmaMembrane
MaUGT174	Malbus0803448.1	459	51.69226	5.63	-0.166	Cytoplasmic
MaUGT175	Malbus0803449.1	456	51.01369	5.39	-0.109	Cytoplasmic
MaUGT176	Malbus0803450.1	475	53.39353	5.55	-0.127	Cytoplasmic
MaUGT177	Malbus0803451.1	452	50.6168	5.21	-0.183	Cytoplasmic
MaUGT178	Malbus0803545.1	468	53.54232	5.56	-0.275	Cytoplasmic
MaUGT179	Malbus0803546.1	497	56.21037	5.77	-0.246	Cytoplasmic

MaUGT180	Malbus0803547.1	468	53.47215	5.41	-0.28	Cytoplasmic
MaUGT181	Malbus0803548.1	471	53.92795	5.65	-0.268	Cytoplasmic
MaUGT182	Malbus0803550.1	499	56.65916	5.88	-0.2	Cytoplasmic
MaUGT183	Malbus0803554.1	473	53.62694	5.3	-0.172	Cytoplasmic
MaUGT184	Malbus0803556.1	487	55.21553	5.59	-0.208	Cytoplasmic
MaUGT185	Malbus0803598.1	484	53.9638	6.13	-0.217	Cytoplasmic
MaUGT186	Malbus0803599.1	480	53.16158	5.86	-0.025	PlasmaMembrane/Cytoplasmic
MaUGT187	Malbus0803601.1	479	53.52161	5.79	-0.101	PlasmaMembrane
MaUGT188	Malbus0803602.1	474	52.85159	5.57	-0.081	PlasmaMembrane
MaUGT189	Malbus0804016.1	465	51.95994	5.95	0.009	PlasmaMembrane

Table S2. Number of *MaUGT* genes in each group according to introns amount.

**No. of Introns / Group**	**0**	**1**	**2**	**3**	**Total**
A	18	3			21
B	2		1		3
D	33	4	2		39
E	45	5	2		52
G		27	5	1	33
H		3		1	4
I		1			1
J		2			2
L	11	12	1		24
M	1	1			2
N		1			1
O	3	2			5
P		2			2
Total	113	63	11	2	189

Table S3. *cis-acting* elements in *MaUGT* promoters.

#ID	G-box	Box 4	AE-box	MRE	LTR	TC-rich	WUN-motif	MBS	ABRE	AuxRR-core	TGA-element	TGACG-motif	CGTCA-motif	GARE-motif	TCA-element	TATC-box	MBSI	circadian	02-site	RY-element	Total
MaUGT 1	0	2	0	0	0	0	0	0	0	0	0	2	2	0	0	0	0	0	0	0	6
MaUGT 2	0	2	2	0	0	0	0	1	0	0	0	1	1	1	1	0	0	0	1	0	10
MaUGT 3	0	4	1	0	1	1	0	0	0	0	2	1	1	0	1	0	0	1	0	0	13
MaUGT 4	0	5	0	0	0	1	0	0	0	0	0	0	0	0	1	0	0	1	0	0	8
MaUGT 5	2	5	2	0	0	0	0	2	2	0	0	2	2	0	1	0	0	0	1	0	19
MaUGT 6	1	2	0	0	1	2	0	0	2	0	0	1	1	0	1	0	0	1	1	0	13
MaUGT 7	2	0	1	1	1	0	0	1	1	0	0	0	0	0	0	1	0	1	1	1	11
MaUGT 8	0	2	0	0	0	0	0	0	0	0	0	2	2	0	1	0	0	0	0	0	7
MaUGT 9	3	0	0	0	0	0	0	1	3	0	1	1	1	1	2	0	0	0	0	0	13
MaUGT 10	2	1	0	0	2	0	1	0	1	0	1	0	0	0	0	0	0	0	0	0	8
MaUGT 11	3	2	0	0	0	0	0	0	2	0	0	2	2	0	0	0	0	0	0	0	11
MaUGT 12	1	0	1	0	0	0	0	1	0	0	0	1	1	0	3	0	0	0	0	0	8
MaUGT 13	1	2	0	0	0	0	0	0	1	0	1	5	5	0	1	0	0	0	0	0	16
MaUGT 14	2	4	0	0	1	0	0	1	1	1	0	1	1	0	1	0	0	0	0	0	13

MaUGT 15	1	2	0	0	0	0	0	0	1	0	0	0	0	0	1	0	0	1	0	0	6
MaUGT 16	2	5	0	2	1	2	0	0	3	0	0	2	2	0	1	2	0	0	2	0	24
MaUGT 17	0	7	0	0	1	3	0	1	0	0	1	0	0	0	0	0	0	0	0	0	13
MaUGT 18	3	3	0	1	0	1	1	1	3	1	0	1	1	0	0	0	0	1	0	0	17
MaUGT 19	1	7	0	2	0	1	0	0	1	0	1	1	1	1	0	0	0	0	2	0	18
MaUGT 20	4	4	1	0	0	0	0	0	3	0	0	0	0	0	1	0	0	0	1	0	14
MaUGT 21	2	3	0	0	3	0	0	0	0	0	1	1	1	0	0	0	0	0	1	0	12
MaUGT 22	4	3	0	1	0	0	0	1	3	0	1	5	5	0	1	0	0	0	2	0	26
MaUGT 23	2	4	0	1	0	0	0	0	1	0	0	3	3	0	0	1	0	0	0	0	15
MaUGT 24	0	3	0	4	0	0	0	0	0	1	0	1	1	1	0	1	0	0	1	0	13
MaUGT 25	1	1	1	0	0	1	0	0	0	0	0	0	0	0	0	0	0	0	1	0	5
MaUGT 26	1	3	1	0	2	0	0	0	1	0	0	0	0	1	1	0	0	1	0	0	11
MaUGT 27	3	4	0	0	1	2	0	0	2	0	2	1	1	0	0	0	0	0	0	0	16
MaUGT 28	0	5	1	0	0	1	0	0	0	0	1	8	8	0	0	0	0	0	0	0	24
MaUGT 29	0	0	0	0	3	1	0	0	1	0	0	0	0	1	0	0	0	0	0	0	6
MaUGT 30	1	1	1	0	0	2	0	0	1	0	0	3	3	0	0	0	0	0	0	0	12
MaUGT 31	1	1	1	1	0	0	0	1	0	0	0	0	0	2	0	0	0	0	0	0	7
MaUGT 32	0	13	0	0	0	0	0	0	0	0	0	0	0	1	2	1	1	0	0	0	18

MaUGT 33	2	4	0	0	1	0	0	0	1	1	3	1	1	0	1	0	0	0	0	0	15
MaUGT 34	3	2	1	1	0	0	0	1	2	0	0	0	0	0	1	0	0	0	1	0	12
MaUGT 35	6	0	0	0	1	1	1	0	4	1	0	1	1	0	4	0	0	1	1	0	22
MaUGT 36	1	4	1	0	1	1	0	0	1	0	1	0	0	0	1	0	0	3	0	0	14
MaUGT 37	4	3	1	0	0	0	0	0	4	0	0	1	1	0	3	0	0	0	0	0	17
MaUGT 38	1	1	0	0	0	0	0	3	1	0	0	2	2	0	0	0	0	0	0	0	10
MaUGT 39	1	0	0	0	1	1	0	1	1	0	1	3	3	0	0	0	1	1	0	0	14
MaUGT 40	3	3	2	0	0	0	0	0	2	0	0	2	2	1	0	1	0	0	1	0	17
MaUGT 41	0	2	0	1	1	3	0	2	0	0	0	0	0	2	0	0	0	0	0	0	11
MaUGT 42	0	6	1	1	1	1	0	4	0	0	2	2	2	0	0	1	0	0	0	0	21
MaUGT 43	2	4	0	0	0	0	0	0	2	1	0	4	4	0	0	1	0	0	0	1	19
MaUGT 44	0	2	0	0	1	1	0	0	0	1	0	2	2	0	1	0	0	1	0	1	12
MaUGT 45	2	2	0	0	0	0	0	0	2	0	0	4	4	0	0	0	0	0	1	0	15
MaUGT 46	4	3	0	0	0	0	0	0	4	0	2	1	1	0	3	0	0	0	0	0	18
MaUGT 47	4	2	0	0	0	3	0	0	3	0	0	3	3	0	2	0	0	0	1	0	21
MaUGT 48	2	3	1	0	0	2	1	0	1	0	0	1	1	1	1	0	0	0	1	0	15
MaUGT 49	1	7	1	0	0	0	0	0	2	0	1	0	0	0	0	0	0	0	0	0	12
MaUGT 50	0	5	0	1	3	0	0	1	0	0	0	1	1	0	0	1	0	0	0	0	13

MaUGT 51	1	0	1	0	0	0	0	2	1	0	0	1	1	1	0	0	0	2	1	0	11
MaUGT 52	3	3	0	1	0	1	0	0	3	0	1	0	0	0	0	0	0	0	0	0	12
MaUGT 53	4	2	2	1	0	0	0	0	4	0	3	0	0	0	0	0	0	0	0	0	16
MaUGT 54	1	0	1	0	0	1	0	0	1	0	3	0	0	0	0	0	0	0	1	0	8
MaUGT 55	0	1	0	1	1	0	0	1	0	0	0	0	0	0	1	0	0	0	0	0	5
MaUGT 56	1	3	1	0	0	0	0	0	1	0	0	1	1	0	0	0	1	0	0	0	9
MaUGT 57	3	3	0	0	1	0	0	0	3	0	0	2	2	0	1	0	0	0	1	0	16
MaUGT 58	2	7	0	0	0	0	0	1	2	0	0	0	0	1	3	0	0	0	0	0	16
MaUGT 59	1	2	0	0	0	0	0	1	1	1	1	0	0	0	2	0	1	0	1	0	11
MaUGT 60	3	1	0	1	1	0	0	1	3	0	1	4	4	0	1	0	0	1	0	0	21
MaUGT 61	2	3	0	0	2	1	0	0	3	0	0	5	5	0	1	0	0	0	2	0	24
MaUGT 62	5	3	0	0	2	0	0	0	5	0	0	1	1	1	1	0	0	0	2	0	21
MaUGT 63	7	5	0	1	0	0	0	0	4	0	1	0	0	0	1	0	0	0	0	0	19
MaUGT 64	0	2	0	0	0	0	0	0	0	0	1	1	1	0	0	0	0	0	0	0	5
MaUGT 65	1	3	0	1	0	1	0	0	1	0	0	0	0	0	1	0	0	1	0	0	9
MaUGT 66	3	4	2	0	0	1	0	1	2	0	0	3	3	0	0	0	0	0	1	0	20
MaUGT 67	0	2	1	0	2	0	0	0	0	1	0	0	0	0	1	0	0	0	2	0	9
MaUGT 68	0	1	0	1	1	0	0	0	0	3	2	3	3	0	1	0	0	1	0	0	16

MaUGT 69	1	2	0	0	1	0	0	0	0	0	0	0	0	0	0	0	0	0	0	0	4
MaUGT 70	2	2	1	0	0	0	0	0	1	0	0	0	0	1	0	1	0	0	0	0	8
MaUGT 71	0	2	0	2	1	0	0	2	0	0	0	1	1	1	0	0	0	0	1	0	11
MaUGT 72	2	0	1	0	3	3	0	0	2	0	1	1	1	0	0	0	0	0	0	0	14
MaUGT 73	2	1	0	0	0	1	0	0	0	0	1	2	2	1	0	0	0	0	1	0	11
MaUGT 74	2	0	1	1	2	2	0	0	1	0	1	0	0	0	1	0	0	2	1	0	14
MaUGT 75	0	2	1	1	1	0	0	1	0	0	0	0	0	0	0	0	0	0	0	0	6
MaUGT 76	4	9	0	0	1	0	0	0	3	0	0	0	0	0	1	0	0	0	0	0	18
MaUGT 77	3	6	0	0	0	0	0	0	2	0	0	1	1	0	0	0	0	0	1	0	14
MaUGT 78	2	3	1	0	1	1	0	2	0	1	0	0	0	0	0	0	0	0	0	0	11
MaUGT 79	2	3	0	2	1	0	1	0	2	0	1	2	2	0	0	0	0	0	0	0	16
MaUGT 80	0	1	1	0	1	3	0	0	1	0	1	0	0	0	1	0	0	0	2	0	11
MaUGT 81	3	3	0	0	0	0	0	0	3	0	2	1	1	0	1	0	0	0	0	0	14
MaUGT 82	0	0	1	1	1	0	0	0	0	0	1	2	2	0	0	0	0	0	0	0	8
MaUGT 83	3	1	0	0	1	0	0	2	2	0	0	2	2	0	2	0	0	1	0	0	16
MaUGT 84	5	0	1	0	0	0	1	0	4	0	0	0	0	0	1	0	0	1	0	0	13
MaUGT 85	2	2	0	0	2	0	0	1	2	0	0	1	1	1	0	1	0	0	1	0	14
MaUGT 86	1	3	0	3	1	0	0	0	1	0	0	1	1	0	1	1	0	1	1	0	15

MaUGT 87	3	2	1	1	0	0	0	1	2	0	1	1	1	0	0	0	0	1	1	0	15
MaUGT 88	2	2	0	0	1	1	0	1	1	0	0	1	1	0	0	0	0	0	0	0	10
MaUGT 89	4	1	0	0	0	0	0	1	4	0	0	1	1	0	0	0	0	0	1	0	13
MaUGT 90	2	2	1	1	1	1	0	1	3	0	1	3	3	0	1	0	0	0	0	0	20
MaUGT 91	3	2	1	1	0	0	0	0	2	0	0	1	1	1	1	0	0	1	0	0	14
MaUGT 92	2	3	0	1	0	0	0	0	1	0	1	1	1	0	0	0	0	0	0	0	10
MaUGT 93	1	3	0	0	0	0	1	0	1	0	1	0	0	0	0	0	0	0	1	0	8
MaUGT 94	1	1	0	0	2	0	0	0	1	0	1	2	2	0	0	0	1	0	1	0	12
MaUGT 95	2	4	1	0	1	0	0	0	1	0	1	3	3	0	1	0	0	0	1	0	18
MaUGT 96	0	3	0	0	0	0	0	0	1	0	1	1	1	0	0	0	1	0	1	0	9
MaUGT 97	6	7	2	0	0	0	0	0	6	0	1	1	1	0	0	0	0	0	0	0	24
MaUGT 98	1	1	0	0	1	1	0	0	1	0	0	0	0	1	0	0	0	1	0	0	7
MaUGT 99	4	3	1	0	0	0	0	1	4	0	0	1	1	1	0	0	0	0	1	0	17
MaUGT 100	1	2	0	0	0	0	0	0	1	0	0	2	2	0	2	1	0	0	0	0	11
MaUGT 101	7	4	0	0	0	0	0	2	6	0	1	1	1	0	2	0	0	0	0	0	24
MaUGT 102	3	1	0	2	0	0	0	1	1	0	0	0	0	0	0	0	0	0	0	0	8
MaUGT 103	0	4	0	1	0	0	0	0	0	0	0	1	1	0	0	0	0	0	0	0	7
MaUGT 104	5	3	1	1	0	1	0	0	4	0	0	3	3	0	0	0	0	0	1	0	22

MaUGT 105	5	6	1	0	0	0	0	0	5	1	1	2	2	0	1	0	0	0	3	0	27
MaUGT 106	9	3	1	1	0	0	0	1	6	0	2	4	4	0	1	0	0	0	1	0	33
MaUGT 107	0	3	1	1	0	1	0	1	0	0	0	0	0	1	2	0	0	0	0	0	10
MaUGT 108	0	1	0	0	0	0	0	1	0	0	0	0	0	0	0	1	0	0	0	0	3
MaUGT 109	0	3	0	0	2	1	0	4	0	1	1	0	0	0	0	1	0	1	1	0	15
MaUGT 110	0	2	1	0	0	1	0	1	0	0	0	2	2	1	2	0	1	0	2	0	15
MaUGT 111	1	2	0	0	0	0	0	1	0	2	0	1	1	0	0	0	0	0	0	0	8
MaUGT 112	1	2	0	0	0	0	0	1	0	2	0	1	1	0	0	0	0	0	0	0	8
MaUGT 113	4	6	0	0	0	1	0	0	3	0	1	0	0	0	1	0	0	0	0	0	16
MaUGT 114	2	2	0	0	1	1	0	0	2	1	1	0	0	0	0	0	0	0	0	0	10
MaUGT 115	0	1	2	0	0	0	0	0	0	0	1	0	0	0	0	0	1	0	0	0	5
MaUGT 116	6	2	1	0	0	0	0	2	7	0	0	0	0	0	2	0	0	1	0	0	21
MaUGT 117	1	3	0	0	0	4	0	1	1	0	0	2	2	0	0	0	0	0	1	0	15
MaUGT 118	1	5	1	0	0	1	0	0	1	0	0	4	4	0	0	0	0	0	0	0	17
MaUGT 119	0	7	0	0	0	1	0	0	0	0	0	1	1	0	0	0	0	0	2	0	12
MaUGT 120	1	1	1	0	1	1	0	0	2	0	0	0	0	0	0	0	0	0	1	0	8
MaUGT 121	1	1	1	0	1	0	0	0	2	0	1	1	1	0	0	0	0	0	1	0	10
MaUGT 122	1	1	1	0	0	1	0	0	2	0	0	0	0	0	0	0	0	0	1	0	7

MaUGT 123	1	1	1	0	0	1	0	0	2	0	0	0	0	0	0	0	0	0	1	0	7
MaUGT 124	9	2	0	0	0	0	0	1	10	0	0	2	2	0	1	0	0	1	1	0	29
MaUGT 125	0	3	0	1	1	2	0	2	0	0	1	3	3	0	1	0	0	0	0	0	17
MaUGT 126	3	4	0	0	0	1	0	1	3	0	0	3	3	0	1	0	0	0	1	0	20
MaUGT 127	0	8	1	0	0	0	0	1	0	0	0	1	1	0	1	0	0	1	0	1	15
MaUGT 128	3	3	0	0	0	0	0	0	3	0	0	1	1	0	0	0	1	0	1	0	13
MaUGT 129	4	3	0	1	0	0	0	1	2	0	0	0	0	1	0	0	0	0	0	0	12
MaUGT 130	1	2	1	1	1	1	1	0	1	0	1	1	1	0	1	0	0	0	0	0	13
MaUGT 131	3	1	1	1	1	2	0	0	1	0	1	0	0	0	1	0	0	0	1	0	13
MaUGT 132	1	0	0	0	0	1	0	1	1	1	1	2	2	1	1	1	0	0	1	0	14
MaUGT 133	2	0	1	0	1	1	0	2	2	0	0	0	0	0	2	1	0	0	0	0	12
MaUGT 134	1	2	0	0	1	0	0	1	1	0	2	0	0	0	1	0	0	0	1	0	10
MaUGT 135	0	3	2	0	1	1	0	0	0	0	0	0	0	0	0	1	0	0	0	0	8
MaUGT 136	3	7	0	0	1	1	0	0	2	0	2	0	0	0	0	0	0	0	0	0	16
MaUGT 137	0	1	1	0	0	0	0	1	0	0	0	0	0	0	1	0	0	0	0	0	4
MaUGT 138	3	4	0	0	1	0	0	0	3	0	1	0	0	0	1	0	0	0	0	0	13
MaUGT 139	0	4	0	0	1	0	0	1	0	1	0	4	4	0	2	0	1	0	0	0	18
MaUGT 140	0	2	0	0	0	0	0	0	0	0	2	0	0	0	0	0	0	0	1	1	6

MaUGT 141	0	0	1	0	1	1	0	0	0	0	0	2	2	0	1	0	0	2	1	0	11
MaUGT 142	4	0	2	0	1	0	0	1	4	0	0	0	0	0	0	0	0	0	2	0	14
MaUGT 143	0	2	0	0	0	1	0	0	0	0	1	0	0	0	0	0	0	1	0	0	5
MaUGT 144	1	3	0	0	0	2	0	0	1	0	0	4	4	0	2	0	0	0	0	1	18
MaUGT 145	0	5	0	0	0	0	0	1	0	0	0	3	3	0	2	0	1	0	0	0	15
MaUGT 146	1	3	0	0	2	0	0	0	1	0	0	0	0	0	1	2	1	0	1	0	12
MaUGT 147	4	1	2	0	0	0	0	1	4	0	0	2	2	0	1	1	0	0	1	0	19
MaUGT 148	19	3	2	1	0	0	0	0	14	0	0	1	1	0	0	0	0	1	0	0	42
MaUGT 149	2	2	0	0	1	3	0	0	2	0	1	0	0	0	0	0	0	0	0	0	11
MaUGT 150	3	1	0	0	0	0	0	3	3	0	0	2	2	1	2	0	0	0	1	1	19
MaUGT 151	1	1	0	0	0	0	0	1	0	0	1	0	0	1	0	0	0	0	1	0	6
MaUGT 152	1	3	0	2	0	0	0	0	0	1	1	0	0	1	2	0	0	0	1	0	12
MaUGT 153	3	1	0	0	1	1	1	1	3	0	0	1	1	1	0	0	0	0	3	0	17
MaUGT 154	3	1	0	0	1	1	1	2	3	0	0	1	1	1	0	0	0	0	3	0	18
MaUGT 155	3	3	0	0	6	1	0	0	2	0	1	0	0	0	0	0	0	1	0	0	17
MaUGT 156	3	1	0	0	2	0	1	0	2	1	0	1	1	0	0	0	0	1	2	0	15
MaUGT 157	4	4	0	0	0	2	0	1	3	0	2	1	1	0	1	0	0	0	1	1	21
MaUGT 158	0	1	2	0	1	0	0	1	0	0	0	1	1	0	1	0	0	0	1	0	9

MaUGT 159	3	3	0	0	1	1	0	1	3	0	0	0	0	0	1	0	0	1	1	0	15
MaUGT 160	3	3	0	0	1	1	0	1	3	0	0	0	0	0	1	0	0	1	1	0	15
MaUGT 161	3	4	0	0	1	1	0	1	3	0	0	0	0	0	1	0	0	1	1	0	16
MaUGT 162	0	2	1	0	0	1	0	0	0	0	0	0	0	0	0	0	0	1	0	0	5
MaUGT 163	3	2	0	0	0	0	0	1	2	0	0	0	0	0	2	0	1	0	1	0	12
MaUGT 164	1	3	0	1	0	1	0	0	1	0	0	1	1	0	0	0	0	0	0	0	9
MaUGT 165	1	5	0	0	0	1	0	0	0	0	0	0	0	0	0	0	0	1	0	0	8
MaUGT 166	1	3	0	0	0	0	0	1	1	0	0	2	2	0	0	0	0	1	2	0	13
MaUGT 167	2	4	1	0	0	0	0	2	1	0	0	1	1	0	1	0	0	1	0	0	14
MaUGT 168	2	7	0	0	0	0	0	0	2	0	1	0	0	0	1	0	0	0	0	0	13
MaUGT 169	2	5	0	1	0	2	0	1	1	0	1	2	2	0	0	0	0	2	0	0	19
MaUGT 170	1	3	0	1	1	0	0	0	1	0	0	0	0	0	0	0	0	2	2	0	11
MaUGT 171	3	2	3	0	2	0	0	0	2	0	0	1	1	0	0	0	1	0	0	0	15
MaUGT 172	0	2	0	2	0	1	0	3	0	0	1	2	2	0	0	0	0	0	1	0	14
MaUGT 173	2	0	0	0	1	1	0	0	0	0	1	3	3	0	0	0	0	0	0	0	11
MaUGT 174	1	0	1	0	0	0	0	1	0	0	0	0	0	1	1	0	0	0	0	0	5
MaUGT 175	2	1	0	0	1	0	0	2	2	0	0	1	1	1	0	1	1	0	1	0	14
MaUGT 176	1	7	2	0	1	2	0	1	1	1	3	1	1	0	1	0	0	1	0	0	23

MaUGT 177	1	3	2	1	0	1	0	1	2	0	0	2	2	1	0	0	0	0	0	0	16
MaUGT 178	1	3	0	0	3	0	0	1	1	0	1	2	2	1	2	1	1	0	0	0	19
MaUGT 179	5	6	0	0	2	0	0	0	4	0	0	2	2	0	1	0	0	0	0	0	22
MaUGT 180	3	4	0	0	1	1	0	0	3	0	0	2	2	0	0	0	0	1	0	0	17
MaUGT 181	3	5	1	0	0	1	0	0	5	0	2	2	2	0	0	0	0	0	3	0	24
MaUGT 182	0	2	1	0	0	0	0	0	0	0	0	1	1	0	2	0	0	0	1	0	8
MaUGT 183	0	4	1	0	0	2	0	0	0	0	1	1	1	1	0	1	0	1	1	0	14
MaUGT 184	3	4	0	0	0	1	0	0	3	0	0	3	3	0	0	0	0	0	0	0	17
MaUGT 185	0	4	0	0	1	1	0	1	0	0	0	2	2	0	0	0	0	0	0	0	11
MaUGT 186	4	4	2	0	0	2	0	0	2	0	1	0	0	1	0	0	0	1	2	0	19
MaUGT 187	0	6	1	0	3	2	0	0	0	0	1	0	0	0	2	1	0	1	2	0	19
MaUGT 188	1	8	1	0	0	1	0	1	0	0	1	3	3	0	2	0	0	0	0	0	21
MaUGT 189	2	0	1	0	0	0	0	0	0	1	1	0	0	0	0	0	0	0	0	0	5
Total	370	531	91	57	109	110	11	102	308	26	93	219	219	38	120	26	16	52	105	8	2611

Supplementary Table S4. Primers used in this study.

Primers	Sequences
UGT29-F	ATCATTCCCTCACTTGGTTCAC
UGT29-R	AGTCTTGTTTGTTGATTGGTGG
UGT68-F	TGAATGCGAGATTATTGACGG
UGT68-R	CCTTACACATTTAGCAACTTCCTC
UGT72-F	AATTCAACCCTTGAGAGTGTAGTG
UGT72-R	CCTTCCATGAGACAAATGCCA
UGT70-F	GTGACAACCCTACAGTGTATCCT
UGT70-R	CTCAATTCCAAACCCAAAGCC
UGT77-F	AACAACTTACTCGTCATAGCCA
UGT77-R	GACCCTCAGTTCCTCCTCAG
UGT156-F	TCAAACAAGCCATCTCTAACC
UGT156-R	CAAACCAAGGAAAGCAACAC
UGT186-F	TTAGGCAAACTCATAGCAACAC
UGT186-R	TGATAGGGAATGAAATGGAAGG
MaUGT68-pYES2-F	cttggtaccgagctcggatccATGAAGGATACTTTAGTTCTATACCCAACT
MaUGT68-pYES2-R	tacatgatgcggccctctagaTTAACGAAGAAGGAAAGGAGAATTT
MaUGT186-pYES2-F	cttggtaccgagctcggatccATGAATAAAAAAGCATGCATTGCT
MaUGT186-pYES2-R	tacatgatgcggccctctagaTCAAAAGTTATACAATTTCAATGCTAAAG
MaUGT68-pET32a-F	gccatggctgatatcggatccATGAAGGATACTTTAGTTCTATACCCAACT
MaUGT68-pET32a-R	ctcgagtgcggccgcaagcttTTAACGAAGAAGGAAAGGAGAATTT
MaUGT186-pET32a-F	gccatggctgatatcggatccATGAATAAAAAAGCATGCATTGCT
MaUGT186-pET32a-R	ctcgagtgcggccgcaagcttTCAAAAGTTATACAATTTCAATGCTAAAG
